# Verbal Ability, Argument Order, and Attitude Formation

**DOI:** 10.3389/fpsyg.2016.01374

**Published:** 2016-09-20

**Authors:** Mindaugas Mozuraitis, Craig G. Chambers, Meredyth Daneman

**Affiliations:** ^1^Analytics and Informatics, Cancer Care Ontario, TorontoON, Canada; ^2^Department of Psychology, University of Toronto Mississauga, MississaugaON, Canada

**Keywords:** verbal ability, argument order, argument integration, attitude formation, persuasion, need for cognition

## Abstract

The current study explored the interaction of verbal ability and presentation order on readers’ attitude formation when presented with two-sided arguments. Participants read arguments for and against compulsory voting and genetic engineering, and attitudes were assessed before and after reading the passages. Participants’ verbal ability was measured, combining vocabulary knowledge and reading comprehension skill. Results suggested that low verbal-ability participants were more persuaded by the most recent set of arguments whereas high verbal-ability participants formed attitudes independent of presentation order. Contrary to previous literature, individual differences in the personality trait *need for cognition* did not interact with presentation order. The results suggest that verbal ability is an important moderator of the effect of presentation order when formulating opinions from complex prose.

## Introduction

Most of the issues considered to be important by modern societies tend to be highly controversial. Using the case of food production and distribution as an example, proponents of genetic engineering might argue that genetically engineered crops and livestock that are nutritious and resistant to disease can play a key role in solving the world’s hunger problems. However, opponents of this view might counterargue that interfering with natural evolution could lead to ecological disasters and might eventually entail even more pronounced global food shortages. People often arrive at a particular viewpoint on the basis of information acquired from written materials such as magazines, newspapers, and books. Thus, it is important to understand how text characteristics as well as individual characteristics of readers jointly contribute to the way in which opinions are formed. Interestingly, although the strength of an argument can play an important role in determining a reader’s response, there is evidence that the order in which arguments are presented can play an important role as well. Further, these text-level features have been argued to interact in specific ways with individual-level traits when readers are forming a particular attitude for a debated topic (e.g., Petty et al., 2001). Of particular interest in the current study is how (a) the *order* in which the arguments are presented, (b) readers’ *verbal abilities*, and (c) readers’ *need for cognition* (an assessment of the general tendency for people to engage in and enjoy effortful information-processing activity) interact in contexts in which individuals formulate opinions about controversial issues presented in text.

A number of reading studies have reported that counterargumentation might have important consequences on learning outcomes (e.g., Braasch et al., 2013; Wolfe et al., 2013). For example, in addition to examining effects related to prior knowledge about a topic, Braasch et al. (2013) explored the role of presenting differing perspectives on a certain physics principle when learning about science. The authors demonstrated that readers were more likely to develop an accurate understanding of the principle of airflow when they were presented with both a popular misconception as well as an explicit and correct refutation, compared to a situation where the correct conception was repeated several times and highlighted as being correct. These findings are consistent with Allen (1991), who found that arguments accompanied by refutations of position-inconsistent views are perceived as more persuasive than arguments that simply present a specific position. This suggests that readers do not simply “navigate past” views and perspectives they ultimately deem to be unpersuasive, but might use them to facilitate learning. One common feature of the studies outlined above is that they focused on the learning of well-established scientific principles. However, many issues important to modern societies, including scientifically grounded ones, can be highly controversial and do not have a single correct answer. In such cases, individuals come to form their own subjective opinions from the information they encounter.

The important role that the ordering of arguments plays in forming one’s attitudes for a given issue was first recognized by Lund (1924). Lund examined how the ordering of pro and con arguments affected attitudes toward issues such as equality in political rights, the implementation of protective tariffs, and monogamy in marriage. Attitudes were measured before participants read the arguments, after they read the first set of arguments (either the pros or the cons), and after they read the remaining arguments (both the pros and cons). Attitude ratings consistently showed that the argument presented first had the largest effect on readers’ final self-reported attitudes about each of the three issues. In view of this pattern, Lund proposed “the law of primacy in persuasion,” holding that arguments presented first in a discussion tend to make a larger impact compared to the arguments presented second. However, some of the subsequent research reported the completely opposite effect (e.g., Cromwell, 1950). For example, Cromwell explored order effects on attitude change with regards to whether the federal government should provide universal medical care and whether the federal government should require arbitration of labor disputes. Cromwell found that when the pro and con arguments were both strong, the view presented second in a discussion had a larger impact compared to the view presented first. When the con and pro arguments regarding the same issue were both weak, order did not have an effect on attitude change. The first attempt to review literature on the topic (Hovland and Mandell, 1957) pointed out that early studies differed on numerous dimensions, including the specific attitude scales used, the timing of probes for reader attitudes, and the perceived relevance of the topics. In addition, given the inconsistent evidence, Hovland and Mandell concluded that the law of primacy does not always apply, and that there are likely other mechanisms that moderate ordering effects when individuals are presented with two conflicting sides of an argument. More recent investigations (outlined in the next sections) have provided new insights about the possible moderators.

One potential moderator of presentation order effects explored in a number of past studies is *need for cognition*, a personality trait that reflects the general tendency for an individual to engage in and enjoy effortful information-processing activity (Cacioppo and Petty, 1982; see also Cacioppo et al., 1996; Petty et al., 2009). Several studies have argued that need for cognition is a reliable moderator of order effects in two-sided argumentation (e.g., Kassin et al., 1990; Haugtvedt and Petty, 1992). More specifically, when presented with two conflicting sides to an issue, individuals with high need-for-cognition scores tend to be more influenced by the viewpoint that was presented first compared to a second viewpoint (a primacy effect), whereas those with low need-for-cognition scores are persuaded more by the more recent viewpoint (a recency effect). For example, Kassin et al. (1990) conducted a mock jury study in which an ambiguous videotaped confession was introduced by one side (defense or prosecution) along with a set of arguments providing an interpretation of the confession as being consistent with that side. Then, the opposing side presented a statement arguing that the confession actually supported the opposite position. Kassin et al. (1990) found that people scoring high in need for cognition gave verdicts based on the initial interpretation of the confession. In contrast, people scoring low in need for cognition relied more on the more recently presented reinterpretation of the confession (see Haugtvedt and Petty, 1992, for similar findings). More recently, Qiu and Wang (2011) investigated how consumers’ attitudes are affected by the presentation order of negative and positive reviews on the Internet. Consistent with Kassin et al.’s (1990) findings, the authors observed that the attitudes of participants scoring high in need for cognition reflected a primacy effect whereas those scoring low in need for cognition showed a recency effect.

The order effects described above have been explained in terms of differences in how low and high need-for-cognition participants process information (Kassin et al., 1990; Haugtvedt and Petty, 1992; Haugtvedt and Wegener, 1994; Petty et al., 2001). Specifically, it is hypothesized that people who score high on need-for-cognition measures are individuals who actively process information and consequently are likely to form opinions early and then engage in confirmatory hypothesis testing. This early opinion is presumed to enable discounting of the later (opposing) arguments. In contrast, people who score low on need-for-cognition measure are individuals believed to engage in less active forms of thinking and, consequently, do not form opinions early. As a result, these individuals are more likely to make decisions only when prompted to do so, and base these decisions on whatever information is most recent and accessible.

An important aspect of the studies to date on this topic is that the arguments are usually presented in complex written or spoken prose. Presumably, the process of formulating attitudes from such materials requires the comprehension and integration of multiple points of view presented in the discourse. A number of authors have investigated how learners combine information from multiple (sometimes contradictory) sources to arrive at a coherent interpretation of a topic (e.g., Wiley et al., 2009; Griffin et al., 2012). For instance, Wiley et al. (2009) evaluated students’ ability to learn about the mechanisms underlying volcanic eruptions. The students were presented with texts from both reliable and unreliable sources. The authors observed that the ability to evaluate the sources was a reliable predictor of the development of an accurate mental model of the phenomena (for similar findings, see Bråten et al., 2009). Furthermore, previous research has established that individuals differ widely in their ability to integrate and comprehend the information in not only a single written or spoken text (Daneman and Carpenter, 1983; Holmes, 1983; Palmer et al., 1985; Perfetti, 1985; Daneman, 1991; Otero and Kintsch, 1992; Hannon and Daneman, 2001) but also multiple documents (e.g., Mason et al., 2010; Griffin et al., 2012). For example, Griffin et al. (2012) explored the effects of individual differences in both capacity and dispositional constraints when learning about a science topic from multiple documents. Following Stanovich and West (1997), the authors reasoned that success on this kind of task reflects individual differences pertaining to both capacity such as working memory, reading skill, and vocabulary knowledge, as well as dispositional characteristics such as an appreciation of the importance of reasoning about evidence when forming and revising beliefs. Griffin et al. (2012) found that for seventh grade students, verbal ability (e.g., teachers’ judgments about a child’s reading skill) was the most reliable predictor of learning outcomes in a multiple-document inquiry task. Although, dispositional characteristics (e.g., scores on the *CLEAR Thinking Scale*) explained a significant proportion of variance in the learning outcomes, the effect was smaller than for verbal ability. Research by Mason et al. (2010) arrived at a similar finding. These authors observed that both reading comprehension skills and beliefs about scientific knowledge were significantly associated with the eighth-graders’ learning about dinosaur extinction from multiple electronic sources. Together these findings highlight that, although dispositional characteristics are relevant when learning from multiple texts, information processing ability might be more important.

Here, we explore the possibility that differences in readers’ verbal ability may also play an important role and perhaps more important than the personality traits such as need for cognition in moderating the effect of argument order when formulating opinions from arguments in written text. For example, individuals with low verbal ability may be less successful at maintaining and integrating successively presented sides for a given argument. As a result they may construct a better representation of the most recent information, and fail to integrate it adequately with the information they were exposed to earlier. This would lead them to rely on the most recently presented position, whether pro or con, when they need to form their attitude toward an issue in question. In contrast, high verbal ability individuals should be better able to integrate the different perspectives and create a more balanced representation of the pros and cons pertaining to a specific issue. Thus, when formulating their opinions they would be able to rely on this balanced representation. In turn, they would be able to form their opinions based on the overall impression of the argument rather than on the most recent position, and so should be generally less susceptible to effects of presentation order in two-sided argumentation. As recognized by Haugtvedt and Petty (1992), although individuals scoring high on need for cognition are more likely to engage in effortful information processing, they are not always inclined to or do not have the required processing ability to do so. Following the same argument, there is no reason for individuals scoring low on need for cognition not to occasionally engage in effortful information processing provided that they have required processing ability to do so. Thus, it is possible that verbal ability may play a more important role than the personality traits such as need for cognition in moderating the effect of argument order when individuals formulate their opinions from arguments in written text.

The present research directly addresses the extent to which readers’ sensitivity to presentation order interacts with individual differences in verbal ability. In addition, it explores how verbal ability relates to need for cognition in view of the previous research addressing the relationship between need for cognition and argument ordering. In the experiment, we manipulate the order in which pro and con arguments were presented to evaluate the effect on readers’ evaluations of these contrasting perspectives. Furthermore, by using both need-for-cognition and verbal ability scores, we provide a novel test of the extent to which individual differences at the level of personality traits and at the level of linguistic-cognitive processing can moderate potential order effects.

Participants were asked to read passages containing four *pro* and four *con* arguments on the topic of compulsory voting for national elections and four *pro* and four *con* arguments on the topic of genetic engineering in food production (see Supplementary Materials for examples). A given participant would read *pros* first for one of the topics and *cons* first for the other topic. In this experiment we also assessed participants’ attitudes toward each topic both before and after they read the arguments. This was done to control for differences in participants’ baseline attitudes toward the selected topics. Furthermore, in this experiment individual differences in verbal ability were assessed by combining scores on the Mill-Hill Vocabulary Scale (Raven, 1965) with those from a global test of reading comprehension ability, the Nelson-Denny (Brown et al., 1981). Individual differences in need for cognition were assessed using a scale developed by Cacioppo and Petty (1982; see also Cacioppo et al., 1984). The scale consists of 18 items where individuals evaluate to what extent statements such as “*I find satisfaction in deliberating hard and for long hours*” serve as accurate descriptions of themselves. Previous research has suggested that individuals scoring high on this scale are naturally inclined to engage in and enjoy effortful information-processing activities involving a wide variety of topics (Cacioppo et al., 1996; Petty et al., 2009).

If low verbal ability individuals are more likely to report the most recently presented position to be the most compelling whereas high verbal ability individuals are, in general, less susceptible to the ordering of arguments, we should observe recency effects for low verbal ability participants and no effect of argument ordering for high verbal ability participants. On the other hand, if need for cognition is indeed a consistent moderator of order effects in two-sided argumentation, the attitudes of participants scoring low on the need-for-cognition measure should be biased toward the arguments presented last (recency effect) whereas the attitudes of participants scoring high on the need-for-cognition measure should be in accordance with the arguments presented first (primacy effect).

## Materials and Methods

### Participants

The participants were 32 female (*n* = 22) and male (*n* = 10) first (*n* = 28), second (*n* = 2), third (*n* = 1) year undergraduate and first year Ph.D. (*n* = 1) students recruited from the University of Toronto Mississauga (mean age: 18.7 years, range: 17–41 years). All participants were fluent speakers of English. Participants were paid $10 (*n* = 1) per hour or received course credit (*n* = 31) for their participation. Participants were tested individually in one session. Each session lasted approximately 1 h. The study was approved by the Social Sciences, Humanities and Education Research Ethics Board at the University of Toronto.

### Pro and Con Arguments

The arguments used in the present experiment were adapted from Sather (1999). The arguments were constructed so that they formed four related pairs of pro and con statements regarding a specific sub-issue to do with compulsory voting and four related pairs of pro and con statements regarding a specific sub-issue to do with genetic engineering (see Supplementary Materials for the full list of arguments used in the current study). In other words, within a given pair, the pro argument addressed an issue for which the con argument served as a direct counterpoint (e.g., compulsory voting ensures that a victorious party represents the views of the entire population vs. if compelled to vote, most people will simply vote randomly, which would not ensure better representation). This point-counterpoint structuring assured that each specific position (whether pro or con) would eventually be complemented by “the other side of the story” that directly addressed the same sub-issue. The length of the sets of arguments related to compulsory voting and genetic engineering was 589 and 565 words, respectively.

### Procedure and Design

Upon arrival each participant was asked to read and sign a consent form. Participants were told the study was designed to examine the ability to learn from text. They were advised that the experimenter would ask some questions about the text when they finished reading.

Each participant read arguments relating to two separate topics (compulsory voting and genetic engineering) and was exposed to both presentation order conditions (between topics: either pro-con then con-pro or con-pro then pro-con). The order in which each topic and each presentation order condition appeared was counterbalanced across participants. Each set of four arguments was presented as a single “page” on a computer screen. An important aspect of this within-participant design is that across the experiment, each set of pro and con arguments appeared in all experimental conditions an equal number of times. In other words, each item with its particular characteristics such as persuasiveness and readability contributed equally to each experimental condition, allowing us to control for these characteristics by the means of the experimental design.

Once participants advanced the page, they were not able to go back (following the methodology used in past studies such as Braasch et al., 2013). Also, participants completed an attitude questionnaire both before and after reading the pro and the con arguments for each topic. Post-reading attitude scores were subtracted from pre-reading attitude scores to derive a single attitude change score. After completing the attitude questionnaire for each topic, participants performed a recall task. Next, participants were asked to complete a prior knowledge questionnaire for each topic as well as the Need for Cognition Scale. The Need for Cognition Scale was administered in the same session following Kassin et al. (1990) and Qiu and Wang (2011). To assess verbal ability, participants completed both the Mill-Hill Vocabulary test and the Nelson-Denny test of reading comprehension (Form E of the Nelson-Denny test: Brown et al., 1981). The Mill-Hill Vocabulary and Nelson-Denny test scores were standardized and averaged to derive a single verbal ability score for each participant.

### Attitude Questionnaire

Participants’ attitudes toward compulsory voting were assessed using nine seven-point semantic differential scales. Following Petty et al. (2001), these scales were labeled *bad*-*good, wise*-*foolish, positive*-*negative, unfavorable*-*favorable, beneficial*-*harmful, unpleasant*-*pleasant, fair*-*unfair, unnecessary*-*necessary*, and *intelligent*-*stupid*. Scales where low numbers indicated positive attitude (e.g., *intelligent-stupid*) were reverse scored so that higher scores always corresponded to more positive attitude. Finally, responses to the scales before and after reading the passages were averaged and subtracted to form one overall attitude change score for each participant.

### Recall Task

Participants were given a booklet for the recall task that stated that the text discussed four issues regarding a respective topic and presented pros and cons for each issue. The instructions then asked participants to write down as much as they could remember about the arguments discussed in the text. The booklet also included a title for each issue (e.g., “Voter turnout,” “Balanced representation,” “Personal liberties,” and “Affirming the privileges for which our predecessors fought” in the case of arguments regarding compulsory voting) on a separate page to serve as memory cues. A single rater blind to the experimental conditions evaluated the responses. The rater assigned a score of 0, 0.25, 0.5, 0.75, or 1 for each of the eight individual arguments depending on how accurate and detailed a given participant’s response was. For example, consider the following con argument regarding compulsory voting:

(1) *Large voter turnout does not automatically ensure that elected officials are representative of the population as a whole. Some people do not vote because they lack interest in the political process. Others may be well-informed, but have no preference for any particular candidate or party. If compelled to vote, these people will vote randomly, which would not ensure that the outcome of the election reflects opinions from all sectors of society.*

To obtain a score of 1 for this particular argument, participants had to indicate four ideas in their answers: (1) some people do not vote due to lack of interest in the political process, (2) some people do not vote due to lack of confidence in the political process, (3) when people who lack interest/confidence vote, they will vote randomly, (4) because votes of disinterested people are random, compulsory voting does not ensure better representation. For each unreported idea, the score was reduced by 0.25. Because each topic as a whole included eight arguments, the highest possible score was 8. In addition, we derived an additional recent-item recall measure by dividing the recall score for the most recent set of arguments out of total recall score. Thus this score measured any bias to recall the information primarily from the most recent set of arguments.

### Need for Cognition Scale

Participants were administered the short version of the Need for Cognition Scale (Cacioppo et al., 1984). The scale includes 18 statements such as “I prefer simple to complex tasks” and “Thinking is not my idea of fun.” Participants were required to rate the extent to which each statement is characteristic of them on a nine-point Likert scale. Half of the statements (e.g., “Thinking is not my idea of fun”) were reverse scored whereas the other half (e.g., “I prefer simple to complex tasks”) were scored regularly. Finally, responses to each statement were averaged to form one overall need-for-cognition score for each participant. Higher scores on this questionnaire corresponded to higher need for cognition.

### Mill-Hill Test of Vocabulary Knowledge

Participants were administered the Mill-Hill test of vocabulary knowledge (Raven, 1965). There were 20 multiple-choice items (e.g., *fecund* means [a] esculent, [b] profound, [c] sublime, [d] optative, [e] prolific, [f] salic). Participants completed all 20 items without any time restrictions. The total number of correctly identified meanings out of 20 was recorded.

### Nelson-Denny Test of Reading Comprehension

Participants were administered the Nelson-Denny test, which consists of eight prose passages and 36 multiple-choice questions. Participants were given 20 min to read the passages and answer the questions. The total number of correctly answered questions within this interval was recorded.

### Prior Knowledge Questionnaire

Participants were then administered a prior knowledge questionnaire to assess how much they knew about the topics of compulsory voting and genetic engineering prior to participating in the study. They were asked two questions: (1) “How much did you know about the idea of compulsory voting/genetic engineering before you came in to do this study?” and (2) “Have you considered the advantages and disadvantages of compulsory voting/genetic engineering before you came in to do this study?” Participants were required to indicate their answer on a seven-point Likert scale (e.g., 1 – “I have never heard about the idea of compulsory voting before” and 7 – “I had a lot of prior knowledge about the idea of compulsory voting”). Responses to each question were averaged together to form participant’s prior knowledge score for each topic.

## Results

### Data Analysis

The recall and attitude change scores were submitted to a series of linear mixed effects models with participants and items as crossed, independent, random effects implemented in package lme4 of the statistical software R 2.15.2 (Bates et al., 2012; R Core Team, 2012; for a discussion on the implementation and advantages of such models over traditional by-participant and by-item analyses based on quasi-*F* tests, see Baayen, 2008; Baayen et al., 2008; Barr et al., 2013). Unlike traditional hierarchical or multilevel models, the mixed effects models applied in the present study do not require random effects to be nested. As suggested by Barr et al. (2013), in all models, we started by including all random effects supported by the design. However, if a maximal model did not converge, the random effects structure was simplified following the “best path” procedure outlined by Barr et al. (2013) until a particular model converged. Main effects and interactions were evaluated performing likelihood ratio tests, in which the deviance (-2LL) of a model containing the fixed effect is compared to another model without the effect in question but is otherwise identical in random effects structure. For the fixed effects, we report the regression coefficient, the standard error, χ^2^ and the corresponding *p-*values (for the verbal ability, need for cognition, recall, recent item recall, prior knowledge, and attitude change score descriptive statistics, see **Table 1**).

**Table 1:** Mean, standard deviation, and ranges for the verbal ability, need for cognition, recall, recent item recall, prior knowledge, and attitude change scores.

Measure	Mean	*SD*	Observed range	Possible range
Pro-con				
Recall				
Pro	1.47	0.75	0.25–3.50	0–4
Con	1.48	0.80	0–3.25	0–4
Total	2.95	1.42	0.25–6	0–8
Recent item recall	0.48	0.14	0–0.71	0–1
Prior knowledge	4.14	1.56	1–6.5	1–7
Attitude				
Pre-reading	4.63	1.16	1.56–7	1–7
Post-reading	4.18	1.29	1.56–7	1–7
Attitude change	-0.45	0.75	-2.67–0.78	-6–6
Con-pro				
Recall				
Pro	1.59	0.82	0.25–3.25	0–4
Con	1.13	0.98	0–3.25	0–4
Total	2.72	1.67	0.25–6.50	0–8
Recent item recall	0.66	0.20	0.33–1	0–1
Prior knowledge	4.01	1.76	1–7	1–7
Attitude				
Pre-reading	4.23	1.15	1–6	1–7
Post-reading	4.28	1.13	1.56–5.78	1–7
Attitude change	0.05	1.07	-3.22–2.89	-6–6
Across conditions				
Nelson-Denny	22.72	4.92	14–34	0–36
Mill-Hill	11.06	2.29	7–15	0–20
Need for cognition	5.84	1.13	3.28–7.72	1–9
Recall				
Pro	1.53	0.78	0.25–3.50	0–4
Con	1.31	0.91	0–3.25	0–4
Total	2.84	1.54	0.25–6.50	0–8
Recent item recall	0.57	0.19	0–1	0–1
Prior knowledge	4.08	1.65	1–7	1–7
Attitude				
Pre-reading	4.43	1.16	1–7	1–7
Post-reading	4.23	1.21	1.56–7	1–7
Attitude change	-0.20	0.95	-3.22–2.89	-6–6

### Association between Individual Differences and Recall

Before turning to the results pertaining to the main focus of the study (attitude formation) we first confirm that our experimental task and the number of participants allows us to replicate more established findings regarding the association between verbal ability, need for cognition, and memory for text information. First of all, we investigated the relationship between the Mill-Hill Vocabulary and Nelson-Denny test scores because they were used to derive a single verbal ability score for each participant. As expected, there was a significant association between the two measures, *r* = 0.49, *p* = 0.005. In addition, consistent with previous research (e.g., Tidwell et al., 2000; Fleischhauer et al., 2010), there was a significant positive correlation between verbal ability and need for cognition measures, *r* = 0.39, *p* = 0.027. The internal consistency coefficient of the Nelson-Denny test was 0.67 (Kuder–Richardson formula 21) and 0.60 (Kuder–Richardson formula 20) for the Mill-Hill Vocabulary test. The internal consistency coefficient of the need-for-cognition scale was 0.89 (Cronbach’s Alpha).

Further, to investigate how the individual differences measures are related to memory, recall scores were first modeled as a function of verbal ability and topic order (whether a set of arguments appeared first or second in the experiment) and the interaction of these two variables (**Table 2**, Model 1). In addition, the model included prior knowledge as a covariate. The analysis revealed that there was a main effect of the order in which the arguments appeared in the experiment, β = 0.53, *SE* = 0.22, χ^2^(1) = 6.05, *p* = 0.014. This indicated that participants recalled more arguments about the topic that appeared second in the experiment (first topic: 2.57 vs. second topic: 3.10). There also was a significant main effect of verbal ability, β = 0.73, *SE* = 0.27, χ^2^(1) = 6.99, *p* = 0.008. This reflects the fact that as verbal ability scores increased, so did recall scores. In addition, the interaction between the two variables was not significant (*p* = 0.788). Finally, there was no main effect of prior knowledge (*p* = 0.979).

**Table 2:** The mixed-effects regression models with Recall as dependent variable.

Effect	β	*SE*	χ^2^ (df)	*p*
Model 1				
Prior Knowledge	<0.01	0.16	<0.01 (1)	0.979
Topic Order	0.53	0.22	6.05 (1)	0.014ˆ*
Verbal Ability	0.73	0.27	6.99 (1)	0.008ˆ*
Topic Order × Verbal Ability	-0.06	0.24	0.07 (1)	0.788
Model 2				
Prior Knowledge	-0.02	0.14	0.02 (1)	0.880
Topic Order	0.52	0.20	6.20 (1)	0.013ˆ*
Need For Cognition	0.18	0.22	0.63 (1)	0.427
Topic Order × Need For Cognition	0.27	0.17	2.42 (1)	0.120
Model 3				
Prior Knowledge	-0.01	0.15	<0.01 (1)	0.965
Topic Order	0.53	0.21	6.54 (1)	0.011ˆ*
Verbal Ability	0.74	0.33	5.46 (1)	0.020ˆ*
Need For Cognition	-0.04	0.24	0.03 (1)	0.855
Verbal Ability × Need For Cognition	0.03	0.33	0.01 (1)	0.923
Topic Order × Verbal Ability	-0.23	0.25	0.93 (1)	0.334
Topic Order × Need For Cognition	0.34	0.19	3.26 (1)	0.071

*^∗^represents significant results at 5% level of significance.*

A model with recall as the dependent measure and need for cognition, the order (first, second) in which a debate topic appeared in the experiment, and their interaction as predictors as well as prior knowledge as a covariate (**Table 2**, Model 2) revealed a significant main effect of debate topic order, β = 0.52, *SE* = 0.20, χ^2^(1) = 6.20, *p* = 0.013. However, there was no main effect of prior knowledge, need for cognition, or an interaction between need for cognition and topic order (*p*s > 0.05).

Next, a model with recall as the dependent measure, prior knowledge as a covariate and verbal ability, need for cognition, topic order, and three two-way interactions between the latter three variables (**Table 2**, Model 3) revealed a significant main effect of debate topic order, β = 0.53, *SE* = 0.21, χ^2^(1) = 6.54, *p* = 0.011. This confirmed that participants recalled more arguments about the topic that appeared second in the experiment and reflects the fact that participants improved at the memory task as they became more familiar with the experiment. There also was a significant main effect of verbal ability, β = 0.74, *SE* = 0.33, χ^2^(1) = 5.46, *p* = 0.020. This reflects the fact that as verbal ability scores increased, so did recall scores even after controlling for need for cognition. However, there was no main effect of prior knowledge, need for cognition, nor were any interactions significant (*p*s > 0.05).

**FIGURE 1:**
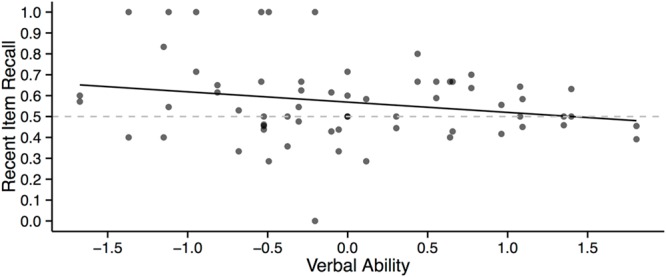
**Relationship between verbal ability and recent item recall.** The proportion scores reflecting recent item recall were logit transformed before entering them into the correlation analysis, however, we plot raw proportion values for easier interpretation.

Recent item recall was also modeled as a function of verbal ability, whether a set of arguments appeared first or second in the experiment, the interaction of these two variables and prior knowledge as a covariate (**Table 3**, Model 1). The analysis revealed that there was a main effect of verbal ability on recent item recall: β = -0.73, *SE* = 0.35, χ^2^(1) = 4.43, *p* = 0.035. As shown in **Figure 1**, as verbal ability increased, the proportion of recent information recalled decreased. In other words, low verbal ability participants tended to recall comparatively more recent information from the presented arguments whereas high verbal ability participants tended to recall information from the earlier and later arguments more equally. Thus, participants with higher verbal ability scores tended to recall the arguments in a more balanced way than did the low verbal ability participants. The model did not reveal any other significant effects (*p*s > 0.05).

**Table 3:** The mixed-effects regression models with Recent Item Recall as dependent variable.

Effect	β	*SE*	χ^2^ (df)	*p*
Model 1				
Prior Knowledge	-0.55	0.40	2.58 (1)	0.108
Topic Order	-0.90	0.63	2.36 (1)	0.125
Verbal Ability	0.73	0.35	4.43 (1)	0.035^∗^
Topic Order × Verbal Ability	0.12	0.70	0.02 (1)	0.895
Model 2				
Prior Knowledge	-0.60	0.41	2.72 (1)	0.099
Topic Order	-0.92	0.66	2.27 (1)	0.132
Need For Cognition	0.06	0.28	0.08 (1)	0.783
Topic Order × Need For Cognition	-0.01	0.55	<0.01 (1)	0.963
Model 3				
Prior Knowledge	-0.63	0.40	3.12 (1)	0.077
Topic Order	-0.93	0.63	2.66 (1)	0.103
Verbal Ability	-0.84	0.41	4.48 (1)	0.034^∗^
Need For Cognition	0.30	0.31	1.10 (1)	0.294
Verbal Ability × Need For Cognition	-0.15	0.42	0.15 (1)	0.699
Topic Order × Verbal Ability	0.13	0.77	0.02 (1)	0.894
Topic Order × Need For Cognition	-0.04	0.59	<0.01 (1)	0.997

*^∗^represents significant results at 5% level of significance.*

In addition, a model with recent item recall as the dependent measure and need for cognition, debate topic order, and Need for Cognition × Topic Order interaction as predictor variables as well as prior knowledge as a covariate (**Table 3**, Model 2) revealed no significant main effects or interactions (*p*s > 0.05). Thus, increases in need for cognition were not significantly associated with changes in the proportion of recent information recalled from the two texts.

Finally, a model with recent item recall as the dependent measure, prior knowledge as a covariate and verbal ability, need for cognition, the order in which a debate topic appeared in the experiment, and three two-way interactions between the latter three variables (**Table 3**, Model 3) revealed a main effect of verbal ability, β = -0.84, *SE* = 0.41, χ^2^(1) = 4.48, *p* = 0.034. This confirmed that the relationship between verbal ability and recent item recall illustrated in **Figure 1** remains significant even after controlling for need for cognition. Finally, no other main effect or interaction was significant (*p*s > 0.05).

Overall, the relationship between the individual differences and the memory measures suggest that verbal ability is more successful than need for cognition at predicting text recall performance (cf. Cacioppo et al., 1996; Kardash and Noel, 2000). Furthermore, the analyses provide evidence that increases in verbal ability are associated with more balanced recall of information presented at the beginning and at the end of each text. This is, however, not the case for the need for cognition measure.

### Individual Differences and Attitudes

The mean attitude change score for all participants was -0.20 (*SD* = 0.57, -0.17, and -0.23 for compulsory voting and genetic engineering topics respectively). This suggests that, overall, participants reported a slightly more negative attitude toward the issues after reading the arguments than they did before reading the arguments, however, this pattern was not statistically significant (*p* > 0.05).

**FIGURE 2:**
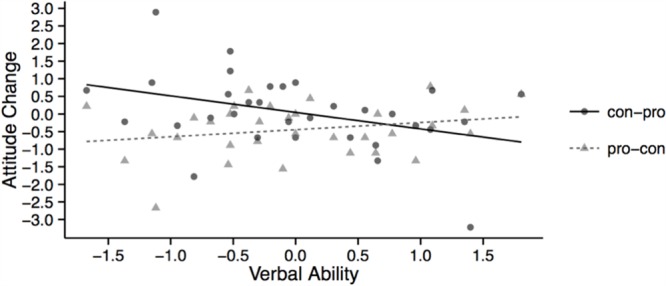
**Illustration of the interaction between presentation order and verbal ability with respect to attitude change**.

To investigate the effect of individual differences and presentation order effects on attitude change, attitude change scores were first submitted to a linear mixed effects model with prior knowledge as covariate and argument presentation order (pro-con vs. con-pro), verbal ability, and Presentation Order × Verbal Ability interaction as predictors (**Table 4**, Model 1). This analysis revealed a significant Presentation Order × Verbal Ability interaction, β = 0.63, *SE* = 0.27, χ^2^(1) = 5.75, *p* = 0.016. To disentangle this interaction, we used the coding of categorical and continuous variables as well as structuring of the model proposed by West et al. (1996; see also Cohen and Cohen, 1983) to test interactions between categorical and continuous variables. As illustrated in **Figure 2**, high verbal ability participants had similar attitude change scores irrespective of argument presentation order, *p* > 0.05. However, low verbal ability participants were more likely to develop more positive attitudes following the reading of the arguments in the con-pro condition compared to the pro-con condition, β = 1.04, *SE* = 0.32, χ^2^(1) = 6.52, *p* = 0.011. In other words, low verbal ability participants showed a recency effect, whereas high verbal ability participants showed neither a recency nor a primacy effect.

**Table 4:** The mixed-effects regression models with Attitude Change as dependent variable.

Effect	β	*SE*	χ^2^ (df)	*p*
Model 1				
Prior Knowledge	-0.09	0.14	0.44 (1)	0.508
Presentation Order	-0.50	0.22	3.83 (1)	0.051
Verbal Ability	-0.13	0.13	1.07 (1)	0.300
Presentation Order × Verbal Ability	0.63	0.27	5.75 (1)	0.016^∗^
Presentation Order When Verbal Ability is High	0.05	0.32	0.02 (1)	0.884
Presentation Order When Verbal Ability is Low	-1.04	0.32	6.52 (1)	0.011^∗^
Model 2				
Prior Knowledge	-0.16	0.14	1.11 (1)	0.293
Presentation Order	-0.49	0.23	3.67 (1)	0.056
Recall	-0.04	0.08	0.32 (1)	0.574
Presentation Order × Recall	0.14	0.16	0.85 (1)	0.356
Model 3				
Prior Knowledge	-0.11	0.15	0.60 (1)	0.438
Presentation Order	-0.56	0.26	3.62 (1)	0.057
Recent Item Recall	-0.03	0.06	0.23 (1)	0.631
Presentation Order × Recent Item Recall	-0.20	0.13	2.34 (1)	0.126
Model 4				
Prior Knowledge	-0.14	0.14	1.00 (1)	0.318
Presentation Order	-0.50	0.23	4.97 (1)	0.026^∗^
Need For Cognition	-0.16	0.10	2.68 (1)	0.101
Presentation Order × Need For Cognition	0.10	0.21	0.24 (1)	0.623
Model 5				
Prior Knowledge	-0.07	0.14	0.24 (1)	0.623
Presentation Order	-0.50	0.22	3.99 (1)	0.046^∗^
Verbal Ability	-0.08	0.15	0.30 (1)	0.582
Need For Cognition	-0.14	0.11	1.67 (1)	0.197
Verbal Ability × Need For Cognition	0.07	0.15	0.24 (1)	0.622
Presentation Order × Verbal Ability	0.69	0.29	6.15 (1)	0.013^∗^
Presentation Order × Need For Cognition	-0.10	0.22	0.23 (1)	0.632

*^∗^represents significant results at 5% level of significance.*

Given that verbal ability was significantly associated with recall and recent item recall, we considered the possibility that these measures might also moderate the effect of presentation order. However, a linear mixed effects model that included prior knowledge, presentation order, recall and Presentation Order × Recall (**Table 4**, Model 2) revealed only a marginal main effect of presentation order, β = 0.49, *SE* = 0.23, χ^2^(1) = 3.67, *p* = 0.056, and no other main effects or interactions. Similarly, a model with prior knowledge, presentation order, recent item recall, and Presentation Order × Recent Item Recall as predictors (**Table 4**, Model 3) also revealed only a marginal main effect of presentation order, β = 0.56, *SE* = 0.26, χ^2^(1) = 3.62, *p* = 0.057. Thus, text memory did not appear to moderate the effect of presentation order in terms of attitude change. We discuss this in more detail in the Section “Discussion.”

In another linear mixed effects model, prior knowledge, presentation order, need for cognition, and Presentation Order × Need For Cognition interactions were entered as predictor variables (**Table 4**, Model 4). The model revealed only a main effect of presentation order, β = 0.50, *SE* = 0.23, χ^2^(1) = 4.97, *p* = 0.026. This reflected the fact that, overall, attitude change was more negative in pro-con than con-pro condition. Importantly, there was no significant Presentation Order × Need For Cognition interaction (*p* > 0.05). Thus, contrary to previous studies (e.g., Kassin et al., 1990; Haugtvedt and Petty, 1992; Qiu and Wang, 2011), we failed to observe different order effects on attitude change for participants that varied as a function of their need-for-cognition scores.

Finally, we modeled attitude change scores as a function of prior knowledge, presentation order, need for cognition, verbal ability, Presentation Order × Need For Cognition, Presentation Order × Verbal Ability, and Verbal Ability × Need For Cognition (**Table 4**, Model 5). The model revealed a main effect of presentation order, β = 0.49, *SE* = 0.22, χ^2^(1) = 3.99, *p* = 0.046. This reflected the fact that, overall, attitude change was more negative in pro-con than con-pro condition. Importantly, the interaction between verbal ability and presentation order illustrated in **Figure 2** remained significant, β = 0.69, *SE* = 0.29, χ^2^(1) = 6.15, *p* = 0.013, even when controlling for the need-for-cognition scores. None of the other main effects or interactions were significant (*p*s > 0.05).

Although we did not find an interaction between presentation order and need for cognition, the results suggest that verbal ability is a significant moderator of primacy and recency effects in two-sided argumentation. More specifically, low verbal ability individuals, perhaps due to their poorer text integration skills, showed a recency effect. However, high verbal ability individuals formed their opinions independent of the argument presentation order. This can be explained on the assumption that high verbal ability participants are able to effectively integrate different ideas presented in the text and rely less on the most recent information when reporting their attitudes. The assertion that high verbal ability participants are more effective at integrating different ideas presented in the text than low verbal ability participants was supported by the significant relationship between verbal ability and recall measures. However, in the current recall task participants were reminded that the text discussed four issues regarding a particular topic and presented pros and cons for each issue as well as indicated a title for each issue. In other words, the recall task provided a structure for the information to be recalled which might not reflect the memory representation of the text at the moment the attitudes were probed. Thus, it may not be entirely surprising that performance on the current recall task did not moderate the effect of presentation order on attitude change.

## Discussion

People often are exposed to differing opinions and viewpoints in written materials such as magazines, newspapers, and books. Research to date has suggested that presentation order as well as need for cognition (or the general tendency for people to engage in effortful information-processing activities) are important factors influencing the way readers deal with these differing viewpoints when formulating their opinions (e.g., Petty et al., 2001). However, in the current study we also examined whether and how verbal ability (individual differences at the cognitive level, relating to the comprehension and integration of text information) may moderate effects of presentation order on attitude formation.

In the current study, we utilized a within-participant presentation order manipulation in which all participants read opposing arguments on two topics—compulsory voting and genetic engineering. For all participants, *pros* were presented first for one of the topics and *cons* were presented first for the other topic. Furthermore, attitudes toward each topic were assessed both before and after reading the sets of pro and con arguments. Consistent with a number of previous studies (e.g., Cacioppo et al., 1986; Kardash and Noel, 2000), verbal ability was a better predictor of text recall than was need for cognition. In addition, as verbal ability scores increased, so did the likelihood that arguments drawn from each of the two opposing positions rather than just the most recent position will be recalled. Together these results demonstrate that readers’ ability to process and comprehend text information is more critical for explaining their ability to encode and/or retrieve this information than their tendency to enjoy information processing. More important, the analyses of the attitude change revealed that there was no interaction between presentation order and need for cognition. Thus, contrary to previous literature (e.g., Kassin et al., 1990; Haugtvedt and Petty, 1992; Qiu and Wang, 2011), in the current study we failed to observe the pattern whereby individuals with high need-for-cognition are influenced more by the first-presented side of an argument compared to the second, whereas those scoring low in need for cognition are persuaded more by the most recent argument.

There are several possibilities for why our findings do not reflect this interaction between presentation order and need for cognition. First, as recognized by Haugtvedt and Petty (1992), although individuals scoring high on need for cognition are more likely to engage in effortful information processing, they are not always inclined to do so. Thus, it is possible that, in the present study, participants scoring high on need for cognition simply did not spontaneously engage in greater information processing. Another possibility is that interaction between presentation order and need for cognition in previous studies was observed due to the specific nature of the pro and con arguments used. In Qiu and Wang (2011), for example, negative and positive reviews were based on different aspects of the product (e.g., short battery life and good appearance, respectively) and were relatively short whereas Haugtvedt and Petty (1992) used arguments that differed in “strength” (e.g., strong vs. weak). In the present study, after reading one set of arguments readers encountered a set of point-for-point counterarguments that directly addressed each of the pros or cons read previously. The symmetry in the structure of the argument may encourage the perception that the last-presented perspective strongly rebuffs the arguments presented first irrespective of the tendency to engage in effortful information processing. Thus, one outstanding question that could be addressed in future research is the extent to which need for cognition is an important moderator of presentation order effects across different kinds of textual materials. Another possibility is that the interaction between need for cognition and presentation order was not observed due to a relatively small sample size employed in the current study. However, it is important to note that this sample was sufficient for replicating a number of previously well-established findings related to associations between the need for cognition, verbal ability, and text recall (for an extensive discussion see the meta-analysis by Cacioppo et al., 1996).

Interestingly, an interaction between presentation order and verbal ability in terms of readers’ attitude change was significant. Specifically, high verbal ability participants had similar attitude change scores irrespective of presentation order whereas low verbal ability participants were more likely to report more positive attitudes following the reading of the arguments in the con-pro condition compared to the pro-con condition (a recency effect). These results suggest that low verbal ability individuals, perhaps due to poor text integration skills and text memory, formed their attitudes based on the most available (recent) information. In contrast, high verbal ability individuals, perhaps due to their superior ability to effectively integrate different ideas presented in text and their superior and more balanced memory for earlier- and later-encountered text, rely equally on earlier and more recent information when reporting their attitudes. This notion is indirectly supported by the observed relationship between the verbal ability and memory measures. However, we do recognize several limitations of the recall task used in the current study. The task probed for recall after participants indicated their attitudes and were reminded that the text discussed four issues regarding compulsory voting and presented pros and cons for each issue along with their titles. Thus, the task provided a type of scaffolding for the information to be recalled after the attitudes were formed. Consequently, the information recalled by the participants in the current task might not straightforwardly reflect the memory representation of the text at the moment the attitudes were formed. This could explain why performance on the current recall measures did not moderate the effect of presentation order on attitude change. One question for future research is whether free recall, which provides less scaffolding, more accurately reflects memory representation of the text at the moment when the attitudes are formed and probed. If so, this recall measure should moderate order effects in attitude formation.

In addition, it is worth noting that readers in the current experiments did not have a possibility to go back to previously read arguments. Hence, it is unclear whether verbal ability would serve as a moderator of presentation order when readers are allowed to selectively reread portions of the text. For example, lower verbal ability individuals may be more likely to engage into rereading and more strategic comparison of the material to compensate for their inferior ability to remember and integrate text information. Furthermore, we recognize that verbal ability is a multidimensional construct involving but not limited to previous experience and working memory capacity. We leave it for further research to explore whether any of these dimensions underlie the observed interaction between verbal ability and presentation order.

Together, the present findings illustrate the importance of incorporating individual differences measures that reflect the ability to understand and integrate ideas presented in a written or spoken discourse into attitude and attitude formation research. Such cognitive skills have received relatively little attention compared to personality attributes such as need for cognition. As observed in the present study, verbal ability was a reliable moderator of presentation order effects on attitude change. We are not suggesting that cognitive ability alone can account for or subsume previously observed effects of personality traits on attitude formation and change. Nevertheless, it is our hope that differences in the ability to understand and integrate ideas presented in a written or spoken discourse will receive the attention they deserve in future research in this area.

## Author Contributions

All authors listed, have made substantial, direct and intellectual contribution to the work, and approved it for publication.

## Conflict of Interest Statement

The authors declare that the research was conducted in the absence of any commercial or financial relationships that could be construed as a potential conflict of interest.
