# Supplementary material for: Verbal Ability, Argument Order, and Attitude Formation
Source: Front Psychol. 2016 Sep 20;7:1374. doi: 10.3389/fpsyg.2016.01374 (PMC5028391; doi:10.3389/fpsyg.2016.01374)
Supplement: Supplementary file 1 [file Data_Sheet_1.DOCX]

Supplementary Material

Verbal ability, argument order, and attitude formation*.*

Mindaugas Mozuraitis*, Craig G. Chambers, Meredyth Daneman

*** Correspondence:** Corresponding Author: email@uni.edu

# Pro and con arguments used in the study*.*

| **Compulsory Voting**  **Pro Argument**  Low voter turnout is dangerous because politicians elected by a minority of voters are not representative of the population as a whole. Because the poor and disadvantaged are far less likely to vote than any other socio-economic group, they can simply be ignored by mainstream politicians. By ensuring that all people cast their vote, compulsory voting guarantees that the outcome of the election reflects opinions from all sectors of society.  It would appear that compulsory voting threatens the right to abstain but voters can still register their dissatisfaction with the choices. For example, if voters do not want to support any given choice, they may spoil their ballot. This is preferred to not voting at all because it ensures there is no possibility that a person has been prevented from voting should they wish to. Thus, voters would still have an opportunity to indicate their dissatisfaction.  Compulsory voting does not restrict personal liberties more than other rules implemented by our society. For example, we still have to pay taxes and follow rules when driving. In addition, the fairness of our legal system depends on compelling people to serve on juries. All of these obligations require far more time and effort than voting does. Thus, compulsory voting can be seen as constituting a much smaller intrusion on liberty than many other activities.  People who fought for universal voting rights made significant sacrifices to ensure that every individual's opinion matters. Their fight and struggles for change within society should not go to waste. We owe it to our ancestors who fought for equality for social groups such as blacks, natives, and women to exercise our democratic right to vote. If people are so apathetic that they will not do this freely, it must be made compulsory.  **Con Argument**  Large voter turnout does not automatically ensure that elected officials are representative of the population as a whole. Some people do not vote because they lack interest in the political process. Others may be well-informed, but have no preference for any particular candidate or party. If compelled to vote, these people will vote randomly, which would not ensure that the outcome of the election reflects opinions from all sectors of society.  Compulsory voting threatens the right to abstain from voting, one of the fundamental principles of contemporary democracies. Just as the right to free speech is complemented by the right to silence, the right to vote is balanced by the right of abstention. Refraining from the voting process is a democratic statement of disenchantment. Forcing those who are unhappy with the state of politics to go and spoil a paper is a pointless waste of resources.  Compulsory voting infringes on an individual’s personal liberty. For example, if people refuse to vote, it can serve as an excuse to fine them or even imprison them. Furthermore, for some social groups, participation in politics is against their religious beliefs. Forcing them to vote explicitly denies them their freedom of religious practice. It is not fair to penalize people or deprive them of their religious practices simply to ensure large voter turnout.  People who fought for universal voting rights sought to make it a right rather than a privilege, but they did not seek to make it an obligation. Campaigners for the rights of blacks, natives, and women have ensured equal access to education and political office, but these groups are not forced to attend university or become a politician. By analogy, although citizens may exercise their right to vote, they should not be compelled to do so.  **Genetic Engineering**  **Pro Argument**  Man has been ‘genetically engineering’ crops and livestock by artificial selection for thousands of years. For example, wheat could never have evolved in the wild and the domestic cat is an artificial animal resulting from 4,000 years of ‘unnatural’ breeding. But in the past the process was indirect and involved painstaking crossbreeding with the goal of keeping certain traits. Genetic engineering simply achieves this process by directly and efficiently manipulating individual genes.  The genetic engineering of plants and animals can have extremely significant benefits for the world’s starving millions. Perhaps the most immediate benefit would be the creation of new crop varieties that are both nutritious and resistant to disease, thereby providing more food without expensive chemical treatments. Developing such varieties would be a boon to the developing world. Thus, genetic engineering has the potential to resolve food shortage problems across the globe.  There is no need to be alarmed about a slight possibility that some dysfunctional varieties may arise due to genetic engineering. First of all, there is no reason why these varieties could have not arisen from traditional crossbreeding. Furthermore, regulations implemented by legislative bodies as well as rigorous industry standards minimize the risk of dysfunctional varieties of crops and livestock reaching farming fields and grocery store shelves.  If applied to humans, genetic engineering can have life-changing medical benefits. For example, a gene could be given to a diabetic to trigger insulin production. Similarly, the genetic material passed onto offspring could be manipulated to remove the genes involved in Parkinson’s disease, breast cancer, and other heritable conditions. Thus, genetic engineering has the potential to eradicate a majority of the diseases that previously could not be cured.  **Con Argument**  Sometimes it is falsely believed that genetic engineering is only a kind of artificial selection. However, genetic engineering introduces genes that are foreign to a species. For example, whereas in crossbreeding we would select two cats possessing desirable traits for mating, genetic engineering involves selecting genes from fish, dogs, and other animals and inserting them into a cat’s DNA. Thus, genetic engineering can have far more dangerous consequences than crossbreeding.  Genetic engineering promises to eliminate global food shortages but completely ignores possible risks. It involves humankind interfering in a domain that should be the preserve of natural evolution. Furthermore, similar promises were made about pesticides around fifty years ago, which proved disastrous for the food chain. The results of tinkering with genes could be even more destructive for the earth’s ecosystem and create food supply problems beyond ones that we are facing today.  Genetic engineering poses a serious risk of developing dysfunctional varieties of crops and livestock. For example, a soybean variety, which had been engineered to resist a particular herbicide, was withdrawn from sale after it was discovered that a brazil nut gene inserted into the soybean caused an allergic reaction in people allergic to nuts. There are no sure safeguards that would prevent these unexpected yet life-threatening outcomes.  Extending genetic engineering to the human genome can have negative medical implications. For example, we know that some genes with negative effects on health such as sickle cell anemia survive because they also bring positive benefits such as immunity from malaria. We do not know how the intricate balance would be affected by permanently altering the genetic make-up of a person. Thus, we should err on the side of caution until our knowledge is better developed. |
| --- |
